# The Effects of Graded Protein Intake in Conjunction with Progressive Resistance Training on Skeletal Muscle Outcomes in Older Adults: A Preliminary Trial

**DOI:** 10.3390/nu14132739

**Published:** 2022-06-30

**Authors:** J. Max Michel, Kristy K. Lievense, Sam C. Norton, Juliana V. Costa, Kathryn H. Alphin, Lydia A. Bailey, Gary D. Miller

**Affiliations:** 1 Department of Health and Exercise Science, Wake Forest University, Winston-Salem, NC 27106, USA; max@alumni.wfu.edu (J.M.M.); lievk20@wfu.edu (K.K.L.); nortsc21@wfu.edu (S.C.N.); jujuvolpini@gmail.com (J.V.C.); kattie413@gmail.com (K.H.A.); bailey.lydia97@gmail.com (L.A.B.); 2 Translational Science Center, Wake Forest University, Winston-Salem, NC 27106, USA

**Keywords:** resistance training, skeletal muscle, aging, protein, hypertrophy

## Abstract

Many studies have evaluated the effects of resistance training (RT) and protein intake to attenuate the age-related loss of skeletal muscle. However, the effects of graded protein intake with conjunctive RT in older adults are unclear. Older adults (*n* = 18) performed 10 weeks of whole-body RT with progressions to intensity and volume while consuming either a constant protein (CP) diet (0.8–1.0 g/kg/d) with no protein supplement or a graded protein (GP) diet progressing from 0.8 g/kg/d at week 1 to 2.2 g/kg/d at week 10 with a whey protein supplement. Data were collected prior to commencement of the RT protocol (PRE), after week 5 (MID), and after week 10 (POST). Dual Energy X-ray Absorptiometry derived lean/soft tissue mass, ultrasonography derived muscle thickness, and a proxy of muscle quality were taken at PRE and POST, while isokinetic dynamometry derived peak torque were taken at PRE, MID, and POST. This study demonstrated the feasibility of the RT protocol (attendance = 96%), and protein intake protocol (CP in range all weeks; GP deviation from prescribed = 7%). Peak torque, muscle quality scores, and appendicular lean/soft tissue mass demonstrated the main effects of time (*p* < 0.05) while no other main effects of time or group * time interactions were seen for any measure. In conclusion, RT improved appendicular lean/soft tissue mass, peak torque, and muscle quality, with no differential effects of graded or constant protein intake.

## 1. Introduction

It has long been accepted that aging is associated with increased physiological dysfunction of many physiological processes [1] including the age-related loss of skeletal muscle mass and quality [2,3]. This age-driven process of skeletal muscle mass and quality loss is implicated in the lean body mass loss-mediated decline in resting energy expenditure [4], increased risk of mortality in disease [5,6], and increased risk of metabolic syndrome, frailty, and insulin response dysfunction [7]. This process is termed sarcopenia, and is characterized by the loss of 3–8% of lean muscle mass per decade after the age of 30 [8]. In an analysis from the Third National Health and Nutrition Examination Survey, it was estimated that up to 59% of women and 45% of men ≥ 60 years of age are classified as sarcopenic [9]. Importantly, however, whether classified as sarcopenic or not, loss of skeletal muscle mass with aging is a debilitating process and attention has been turned to the development of pragmatic interventions.

In particular, resistance training (RT) has been the focus of many interventions in hopes to attenuate or reverse the age-related decline in skeletal muscle mass. In many instances, RT has been proven an effective intervention to combat the effects of aging on skeletal muscle [10,11,12,13]. Indeed, studies and meta-analyses in populations aged ≥50 years have found significant increases to lean mass and muscle morphology measures [10,14,15,16]. RT has additionally been shown to increase strength outcomes and muscle activation in older adults [16,17,18]. Many interventions in older individuals have found significant strength increases as measured by one repetition maximum (RM) testing, isokinetic dynamometry, and isometric dynamometry [19,20,21,22].

While these results are promising, such adaptations in older adults typically do not occur to the same degree as their younger counterparts [20]. It has been posited that this difference in magnitude of response is due to a phenomenon termed anabolic resistance, or the reduction in muscle protein synthesis in response to an anabolic stimulus (e.g., resistance training) [19]. In order to combat this, the combination of enhanced protein intake and RT has been the subject of attention. Indeed, it has been suggested for older adults performing RT that acutely, ≥40 g of protein intake in a single bolus may be optimal [23,24,25,26], while chronically intake of ~1.6 g of protein per kg of bodyweight per day (g/kg/d) might provide maximal benefit as demonstrated in a breakpoint analysis [27]. Notably, in this aforementioned analysis the 95% confidence interval ranged from 1.03 to 2.20 g/kg/d. Furthermore, Antonio et al. have demonstrated that chronic protein intakes > 3 g/kg/d demonstrate no adverse effects in a one-year crossover study [28]. Interventions adopting strategies comparable to these have shown promising results, as meta-analyses have found that protein supplementation augments fat free mass gain [29] and strength measures [27,30] in older adults as compared to RT alone. There are, however, conflicting findings, as several studies found no benefit to enhanced protein intake and/or protein supplementation when combined with RT in regard to body composition and/or muscle morphology [14,21,31,32], and strength measures [33,34,35].

It stands to reason that increasing protein intake concurrently with training intensity/volume could enhance skeletal muscle outcomes given that (a) large single protein boluses (≥40 g) have proven beneficial for older adults, (b) 1.6 g/kg/d has proven more effective than the RDA of 0.8 g/kg/d for adults undergoing RT, and (c) increased protein intake, specifically enhanced leucine intake, has shown promising effects in older adults at the cell signaling level [19,36,37,38].

While many studies have examined the effects of enhanced protein intake in conjunction with RT on skeletal muscle adaptation in older adults, none have adopted the strategy of grading protein intake to RT intensity and volume. Therefore, the purpose of the present study was to examine the feasibility and effects of graded protein intake in conjunction with RT while maintaining both large single protein boluses and average sustained intake of ~1.6 g/kg/d as compared to constant protein intake in conjunction with RT on skeletal muscle outcomes in older adults. The primary outcome of this study was change in overall and appendicular lean/soft tissue mass. Secondary aims of this study were change in vastus lateralis thickness, muscle quality, isokinetic dynamometry derived strength measures, and lean/soft tissue mass index. We additionally aimed to assess the feasibility of both the RT and nutritional protocols, defined as at least 80% attendance to training sessions and deviation of no more than 20% from target protein intakes. We primarily hypothesized that graded protein intake with RT would produce greater total and appendicular lean/soft tissue mass gain than would constant protein intake with RT. We additionally hypothesized that graded protein intake with RT would provide greater adaptation to vastus lateralis tissue thickness, muscle quality scores, and strength measures than would constant protein intake with RT. Finally, we set out to examine the feasibility of the RT and nutrition protocols amongst participants.

## 2. Materials and Methods

### 2.1. Participants

This study was approved by the Institutional Review Board at Wake Forest University Reynolda Campus and conformed to standards set by the latest revision of the Declaration of Helsinki (IRB Approval No. IRB00024112). This trial was registered as a clinical trial at www.clinicaltrials.gov (ID: NCT04845282), accessed on 27 April 2022. Healthy, community-dwelling older men and post-menopausal women (≥55 years) were recruited to participate in this study. Participants provided verbal and written informed consent and were screened prior to study initiation. Participants were required to be free from comorbidities that could be exacerbated by study protocols such as: cardiovascular disease, type 1 or type 2 diabetes, renal failure, or thyroid disorders; or were required to provide explicit written consent from a physician stating that they were medically cleared to participate in the study after review of study protocols by the participant’s primary care physician. Participants were excluded if they were: currently consuming an agent known to be confounding to skeletal muscle adaptation, used a whey protein supplement regularly over the previous three months, or if they had adhered to a progressive RT program in the 3 months prior to study initiation. A progressive RT program was defined as a program primarily consisting of RT wherein volume, intensity, frequency, or overall difficulty were monitored and modulated. Prior to study initiation, participants were instructed to cease any other RT activities outside of study protocols. Importantly, participants were instructed to refrain from external RT, or the use of any sort of protein supplement other than what was prescribed by study protocols (GP only).

### 2.2. Study Design

**Figure 1:**
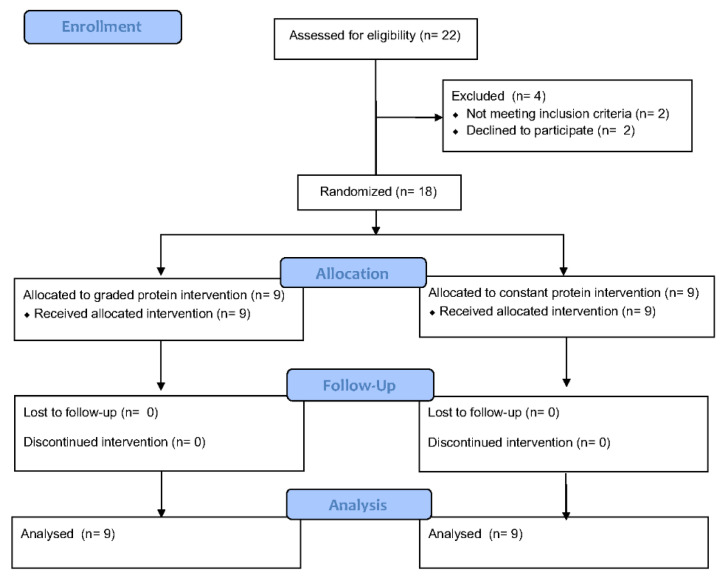
Study Consort Diagram.

This was a two-arm, 10-week randomized clinical trial. Study design is depicted visually in Figure 1 and Table 1. Briefly, prior to randomization, interested individuals gave consent and underwent baseline testing. After screening and baseline data collection, 18 participants were randomly assigned to one of two groups: graded protein (GP) and constant protein (CP). Select measures were obtained after week 5 (MID) and the full testing battery was taken after completion of week 10 (POST). Measures and descriptions of the intervention groups are provided below.

#### 2.2.1. Resistance Training Protocol

After baseline data collection, participants familiarized themselves to equipment and movements, and completed 3 RM testing to derive an estimated 1 RM that was used for intensity prescription throughout the study. The 3 RM testing was conducted in accordance with National Strength and Conditioning Association protocols [39]. Following the acclimation and 3 RM testing week, participants performed 3 lower extremity-focused, full-body RT sessions per week at Wake Forest University’s Department of Health and Exercise Science Clinical Research Center (CRC). RT protocols were in accordance with the American College of Sports Medicine’s 2009 position stand, “Progression models in resistance training for healthy adults” [40]. The RT progression model is depicted in Table 1.

Briefly, training intensities began at 60% predicted 1 RM at week 1 and progressed to 75% 1 RM by week 4, after which participants underwent a deload week (50% 1 RM), or an intentional drop in intensity and volume, to potentiate recovery and performance for subsequent training and testing. After completing the deload week, the second 5 week training block commenced at 70% 1 RM and progressed to 85% 1 RM by week 9. Participants again underwent a deload week prior to post-testing. Participants were instructed to complete 8–12 repetitions per set per exercise. If this repetition range was unattainable for a participant, intensity was decreased by 5% per repetition missed. Exercises completed in each session were: Session 1: leg press, machine incline chest press, compound machine row, machine triceps pressdown, leg extension; Session 2: machine chest press, machine hamstring curl, machine calf raise, machine bicep curl, leg press; Session 3: leg extension, machine overhead press, compound machine row, machine triceps pressdown, leg press. These exercises were chosen based on their common inclusion into RT programs as well as availability at the CRC. Adherence to training session was monitored throughout the study, and defined as training sessions attended divided by training sessions prescribed. All training sessions were overseen by an investigator in order to ensure proper technique.

#### 2.2.2. Nutrition Protocol

The present nutrition modification intervention commenced at week 1 after the acclimation and 3 RM testing week. Participants were randomly assigned to either GP or CP, and protein intake levels differed between groups. Protein intake levels by week for each group are depicted below in Table 1. Briefly, the GP group began at 0.8 g of protein per kg of bodyweight per day (g/kg/d) from both a whey protein supplement and dietary sources. Protein intake subsequently increased throughout the course of the study to 2.2 g/kg/d at weeks 9 and 10. Notably, protein intake levels did not increase for deload weeks (week 5, week 10) due to training intensity and volume decreasing. The protein intake strategy was chosen because (a) the targeted mean protein intake across 10 weeks was 1.56 g/kg/d, very close to the value of 1.6 g/kg/d recommended by Morton et al. [27], (b) by increasing protein intake to 2.2 g/kg/d at weeks 9 and 10 the full 95% confidence interval presented by Morton et al. was consumed by participants, and (c) high protein intakes > 2.0 g/kg/d have previously been observed to be safe and efficacious in older adults [41,42]. The whey protein supplement, Combat 100% Whey (MusclePharm^®^, Las Vegas, NV, USA), was provided to this group to assist in achieving protein intake goals. Whey protein was chosen based on its high leucine content as compared to other protein supplements [43,44]. Additionally, supplementation was provided as one serving (25 g) per day in weeks 1–5 after which it increased to two servings (50 g) per day at week 6. In the weeks thereafter (weeks 7–10), protein supplementation could be increased to 75 g per day at any time by either choice of the participant, or failure to meet protein intake goals through the diet. For protein supplement boluses up to 50 g, all protein was consumed immediately after training on training days, and between meals on non-training days. If protein supplement intake reached 75 g, participants were instructed to take 50 g immediately post-training and 25 g between meals on training days, and at two separate occasions in between meals on non-training days. Notably, on weeks in which protein supplementation remained constant and overall protein prescription increased, participants were instructed to increase dietary protein intake (e.g., weeks 1–5 where protein intake targets increased from 0.8 to 1.4 g/kg/d while supplemental protein was held constant at 25 g/d).

Conversely, the CP group was instructed to consume protein at a constant level of 0.8–1.0 g/kg/d, in accordance with the recommended daily allowance, for the duration of the study. This group was additionally not provided a protein supplement. Adherence to prescribed protein intake levels was monitored by study staff throughout the study for both groups. Additionally, both groups were prescribed a caloric surplus of 200–300 kcal based on the Harris-Benedict equation using the moderate activity factor in order to potentiate skeletal muscle hypertrophy [45]. Participants were also instructed to consume 3–5 g of carbohydrates per day based on recommendations from Slater and Phillips [46]. Fats made up the remainder of calories for a given day. A heavy emphasis was placed on protein intake levels, as this was the differentiating intervention strategy between groups. Nutrient intake was tracked via a mobile application (MyFitnessPal, Inc.; Baltimore, MD, USA) that has been validated against paper-based food records [47]. Investigators maintained access to each participants dietary entries for the duration of the study, allowing for collection and analysis of data. Participants were asked to track food intake 3 days per week (2 weekdays and 1 weekend day), a strategy that has been used previously [48]. Nutrient intake was monitored weekly by study staff, and if participants were out of the desired range for protein they were instructed on how to adjust intake the following week to meet their goals. Overall target protein intakes by group are presented in Table 1 below.

### 2.3. Measures

As outlined in Table 1, the following measures were taken prior to (PRE), during (MID) and after (POST) the 10 week RT protocol. Participants were instructed to arrive to testing sessions involving imaging (PRE and POST) in an overnight fasted state, approximately 48–72 h after their most recent training session (POST) or exercise (PRE).

#### 2.3.1. Height, Weight, and Body Mass Index

Height was measured using a wall-mounted Seca 216 stadiometer (Seca; Hamburg, Germany) at PRE. Participants were instructed to remove their shoes and stand with their back to the wall with eyes facing straight in front. The measuring bracket was then pulled down until it laid flat against the head of participants. Height was recorded to the nearest 0.5 cm. Weight was measured at all testing sessions using a Tanita scale (Tanita; Arlington Heights, IL, USA) after the removal of all outerwear and shoes. Weight was recorded to the nearest 0.1 kg. Body Mass Index (BMI) was calculated by using the CDC promoted equation of weight in kg/height in m^2^.

#### 2.3.2. Lean/Soft Tissue Mass

Whole-body and regional lean/soft tissue mass were determined via dual energy X-ray absorptiometry (DXA) scan at both PRE and POST. Participants were instructed to perform an overnight fast and were then subjected to total-body DXA testing (GE Lunar iDXA; GE Corporation, Fairfield, CT, USA). The DXA was calibrated immediately prior to each testing session and passed all quality checks. Participants were instructed to wear clothing free of any metal; if this instruction was violated, participants were provided standard hospital gowns. All metal objects (i.e., jewelry), shoes, and outer clothing were then removed, and participants were instructed to lay supine with palms down in the field of view of the machine by the same trained and experienced research staff at all time points. After participants were positioned, the DXA scan commenced and lasted approximately 10 min per participant. Regions of interest were set to create accurate regional measurements and underwent quality control adjustments by a departmental certified bone densitometry technologist. The DXA system in our department has a coefficient of variation of 0.85% for measures of lean/soft tissue mass. Notably, both total body and appendicular lean/soft tissue mass were collected and subject to analysis. Appendicular lean/soft tissue mass was defined as the combined value of lean/soft tissue mass from the arm and leg regions.

#### 2.3.3. Muscle Tissue Thickness

Following body composition testing, participants were tested for vastus lateralis thickness using ultrasound at PRE and POST. Vastus lateralis thickness was determined by placing a 13–6 MHz transducer (SonoSite M-Turbo; FUJIFILM Corporation, Minato City, Tokyo, Japan) midway between the inguinal crease and the superior aspect of the patella. Measurements were taken from the supine position after ≥10 min to account for fluid shifting. Measurements were taken with accompanying software and were made by aligning the measuring calipers with the outer connective tissue and the inner connective tissue, thus surrounding the vastus lateralis. Measurements were taken immediately after image capture and saved according to manufacturer protocols. All images and measurements were taken by the same investigator in order to minimize intertester variability among measurements as suggested previously [49,50]. This investigator (JMM) possessed an intraclass correlation coefficient of 0.994 as determined by a test-retest protocol on a subset of 10 participants.

#### 2.3.4. Peak Torque

Knee extensor peak torque was assessed with the use of an isokinetic dynamometer (Humac Norm; Computer Sports Medicine Incorporated, Stoughton, MA, USA). The subject’s dominant leg was tested at PRE, MID, and POST at 60°/s and 120°/s moving through a 30° range of motion. Upon entry, the subject was informed of protocols and the purpose for the test. Participant anthropometric information was entered into the accompanying software after which the dynamometer was adjusted to software derived recommendations. The rotational axis was then aligned with the lateral epicondyle of the subject’s dominant leg and the testing protocol began. Participants performed 3 repetitions of concentric extension and flexion at both 60°/s and 120°/s to practice the motion that would be required for the test. After practice repetitions were complete, participants completed 5 repetitions at 60°/s and 120°/s respectively and the peak torque achieved over 5 repetitions was calculated by the dynamometer. After the test was complete, data were saved and exported for analysis.

#### 2.3.5. Skeletal Muscle Quality and Skeletal Muscle Index

Given that muscle quality has been defined as strength per unit area of muscle, a proxy measure of skeletal muscle quality has previously been defined as a strength measure divided by lean mass of the area of interest [51]. Given the particular interest in skeletal muscle quality in older adults, we attempted to measure skeletal muscle quality as knee extensor peak torque /leg lean mass at a given time point (PRE and POST). We additionally used DXA derived lean/soft tissue mass to determine lean mass index, defined as lean/soft tissue mass in kg/height in m^2^ at PRE and POST.

### 2.4. Analytic Plan

All data were initially checked for normality using a Shapiro–Wilk test set at a significance level of *p* ≤ 0.05. If data were normally distributed, parametric techniques were used. If data were not normally distributed, a square-root transformation was performed. If data were then normally distributed parametric techniques were used. For analyses using repeated measures analysis of variance (ANOVA), data were tested for assumptions of sphericity by using Mauchly’s test of sphericity at a significance level of *p* ≤ 0.05. For those data sets where sphericity was violated, Greenhouse–Geisser corrections were used. Independent samples t-tests were additionally performed across all measures at baseline to ensure no significant differences at baseline. A significance level of *p* ≤ 0.05 was adopted for all analyses. To test measures with 2 timepoints (PRE, POST) (Lean/Soft Tissue Mass, Muscle Tissue Thickness, Muscle Quality Score, Skeletal Muscle Index) a 2 × 2 (group * time) ANOVA was performed to examine the main effects of group and time as well as group * time interaction. Measures taken at 3 timepoints (PRE, MID, POST) (peak torque) were analyzed using a 2 × 3 (group * time) ANOVA. These data were tested for the main effects of group and time as well as for group * time interaction. For all of the statistical models described above, if a significant group * time interaction was present, data were tested for the simple main effects of group and time with Bonferonni post hoc comparisons. Additionally, Bonferroni post hoc comparisons were generated in the absence of a significant group * time interaction to compare the main effects of group or time. All analyses were performed using IBM SPSS v28.0 (Chicago, IL, USA). In addition to formal statistical analyses presented above, post hoc power analyses were conducted for all measures in G * Power v3.1 to present a summary of effects. 

## 3. Results

### 3.1. Participants 

Participants did not differ significantly in any baseline descriptive characteristics; these data are presented in Table 2. Mean age for participants was 69.7 ± 8.2 years. Both the GP and the CP had the same number of participants (*n* = 9), with the CP group having three males and six females and the GP group having four males and five females.

### 3.2. Self-Reported Dietary Intake

Nutritional analyses were performed on all participants, given all 18 participants logged ≥90% of requisite inputs. There was a significant group * time interaction as well as a main effect of time for both absolute (kcal/day) and relative (kcal/kg/day) energy intake (*p* < 0.001), where the GP group increased kcal intake over time, and the CP group did not significantly increase energy intake over the course of the study. There was a main effect of group for relative (*p* = 0.010), but not for absolute energy intake (*p* = 0.096). These data are presented in Table 3.

By study design, there were the main effects of group and time as well as a significant group * time interaction for both absolute (g/day) and relative (g/kg/day) protein intake (*p* < 0.001). These data are presented in Table 4. The GP group exceeded prescribed protein intake for weeks 1–2 by 37.92% and 15.10% respectively, after which this group deviated no more than ~5%. Target vs. actual protein intake values for the GP group are presented in Table 5. The CP group fell within the prescribed protein intake range for all 10 weeks of the intervention. Protein supplement adherence in the GP group was 81% as determined via bag return (supplement bags returned/supplement bags distributed * 100).

### 3.3. Training Volume and Attendance

**Figure 2:**
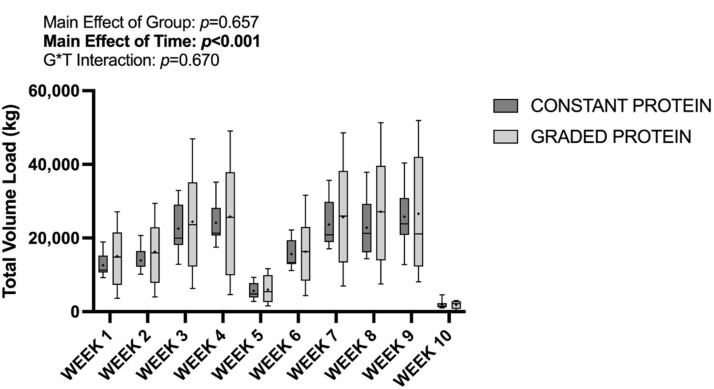
Resistance training total volume load (reps * sets * load). G*T: group * time; blod font: significant main effect.

By study design, there was a significant main effect of time observed for overall training total volume load (total reps * sets * load) (*p* < 0.001), with no significant group * time interaction (*p* = 0.653), nor a significant main effect of group (*p* = 0.631). Training session attendance was 98% overall, and did not differ between groups at any week (*p* = 0.286). These data are presented in Figure 2 below.

### 3.4. Lean/Soft Tissue Mass and Lean/Soft Tissue Mass Index

**Figure 3:**
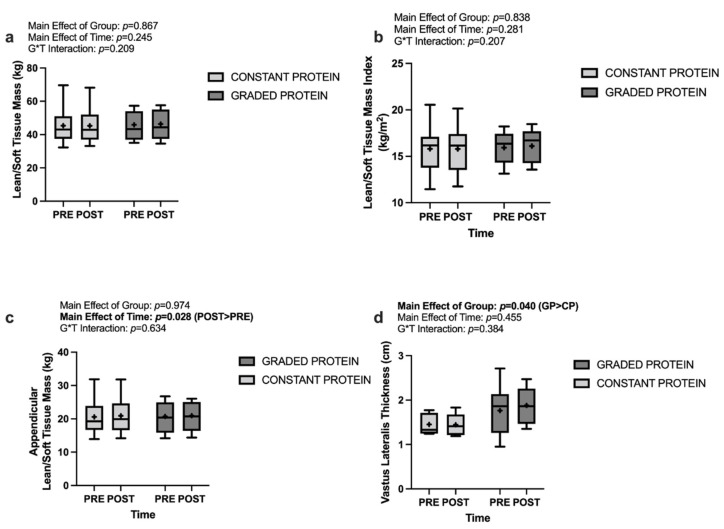
Changes in muscular variables between groups and across time for (**a**) lean/soft tissue mass, (**b**) lean/soft tissue mass index, (**c**) appendicular lean/soft tissue mass, (**d**) vastus lateralis thickness. blod font: significant main effect.

Analyses of overall lean/soft tissue mass and lean/soft tissue mass index did not demonstrate a significant group * time interaction, a significant main effect of time, or a significant main effect of group. Data are presented in greater detail in Figure 3a,b. Analyses of appendicular lean/soft tissue mass revealed no significant group * time interaction (*p* = 0.634) nor a significant main effect of group (*p* = 0.974), but did reveal a significant main effect of time (*p* = 0.028) with POST being greater than PRE by 0.269 kg across both groups. These data are presented in Figure 3c.

### 3.5. Vastus Lateralis Thickness

Analyses of ultrasonography derived vastus lateralis thickness did not indicate a significant main effect of time (*p* = 0.455) or a significant group * time interaction (*p* = 0.384). These analyses did, however, demonstrate a significant main effect of group (*p* = 0.040), with GP having a larger mean vastus lateralis thickness by 0.373 cm. These data are presented in Figure 3d.

### 3.6. Peak Torque

**Figure 4:**
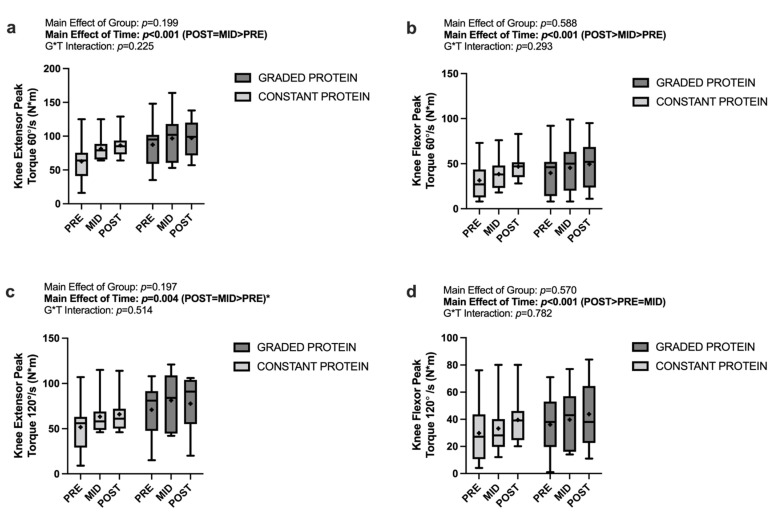
Changes in strength measures between group and across time for (**a**) knee extensor peak torque at 60°/s, (**b**) knee flexor peak torque at 60°/s, (**c**) knee extensor peak torque at 120°/s, (**d**) knee flexor peak torque at 120°/s. * POST not significantly different from PRE or MID; blod font: significant main effect.

Analyses of knee extensor and flexor peak torque at both 60°/s and 120°/s demonstrated the significant main effects of time (*p* ≤ 0.004) with values increasing over time. There were no significant main effects of group (*p* ≤ 0.197) nor any significant group * time interactions (*p* ≤ 0.225). These data are presented in Figure 4a–d.

### 3.7. Muscle Quality Score

**Figure 5:**
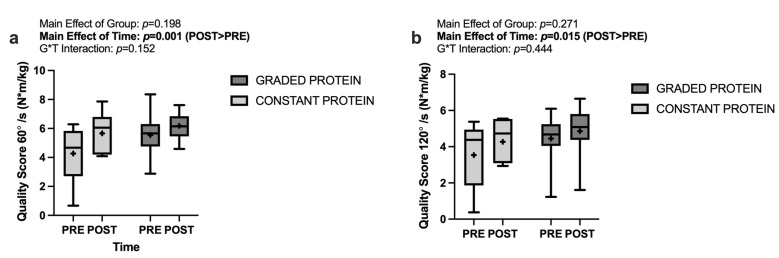
Changes in muscle quality score between groups and across time at (**a**) 60°/s, and (**b**) 120°/s. blod font: significant main effect.

Analyses of muscle quality score (knee extensor peak torque values divided by leg lean mass at the corresponding time point) at both 60°/s and 120°/s demonstrated the significant main effects of time (*p* ≤ 0.015) with POST being greater than PRE by 1.011 N·m/kg at 60°/s and 0.676 N·m/kg at 120°/s. There were no significant main effects of group (*p* ≥ 0.198) nor any significant group * time interactions (*p* ≥ 0.152). These data are presented in Figure 5a,b.

### 3.8. Analysis of Effects

An overall analysis of effects was performed as a part of this study. *p*-values, F-values, η_p_^2^, observed power, and estimated sample size are presented for each outcome measure in Table 6 below.

## 4. Discussion

The purpose of the present study was to examine the effects of graded protein intake vs. constant protein intake on skeletal muscle adaptations in older adults undergoing RT. The main findings of this study include: (i) appendicular lean/soft tissue mass improved across time; (ii) vastus lateralis thickness was significantly higher in the GP group than the CP group; (iii) muscle quality scores improved across time at two different knee extension velocities; (iv) all strength measures improved across time; (v) there were no significant between group improvements or group * time interactions for total body or appendicular lean/soft tissue mass; (vi) the protein intake and RT protocol were feasible among both groups. The primary hypotheses that the GP group would realize greater adaptation to total body and appendicular lean/soft tissue mass than the CP group were not supported. The secondary hypotheses that the GP group would have greater increases in vastus lateralis thickness, peak torque, muscle quality score, and lean/soft tissue mass index to a greater degree than the CP group were also not supported. However, it was observed that both the RT and graded protein protocols were feasible in a cohort of older adults. 

### 4.1. Nutrition and Resistance Training Intervention

Based on self-reported dietary records, the CP group remained in desired range of protein intake for the entire 10-week intervention, and the GP group deviated no more than 5.4% after week 2 of the intervention. This demonstrates the feasibility of the dietary protein intervention. Additionally, overall average self-reported protein intakes per group as compared to prescribed were 1.59 g/kg/d (actual) vs. 1.56 g/kg/d (prescribed) for the GP group and 0.96 g/kg/d (actual) vs. 0.8–1.0 (prescribed) for the CP group. Supplementation adherence was 81% measured via bag return. Self-reported total energy consumption was lower than prescribed. The literature regarding self-reported dietary intake consistently demonstrates that self-reported energy intake underreports true energy intake [52,53,54]. Despite the limitations of self-reported dietary intake, the results from the present study indicate that the proposed protein intake paradigm is feasible in a population of older adults.

### 4.2. Lean/Soft Tissue Mass and Vastus Lateralis Thickness

While appendicular lean/soft tissue mass improved ubiquitously over time, overall lean/soft tissue mass did not demonstrate any significant changes. This perhaps stands to reason given that the primary focus of the training intervention was the lower body. The lack of difference between groups is supported by findings where an increase in appendicular lean/soft tissue mass has been seen in both protein intake modification [55,56] and RT [57] interventions alike. Indeed, even in a cohort of young adults, it has been seen that extremely high-volume RT increased full-body and appendicular lean body mass similarly regardless of supplementation [58]. Gain in appendicular lean/soft tissue mass is of particular interest given its role in maintaining proper locomotion and general functionality in older adults. Indeed, appendicular lean mass has been associated with higher dynamic balance scores [59] and a 50% lower risk of all-cause mortality [60] in older adults. While total body lean/soft tissue mass did not exhibit significant changes, it is possible that in an increased study period, gains in appendicular lean/soft tissue mass could have proved robust enough to influence overall lean/soft tissue mass values.

Lack of overall gain in lean/soft tissue mass has been observed previously in RT interventions ranging from 6 to 12 weeks [61,62,63,64]. One potential contributing factor to this finding is deficits between self-reported vs. prescribed energy intake. Previous studies have found that energy deficits of ~500 kcal/day [65] and ~600 kcal/day [66] impaired lean mass gain in older adults undergoing RT. Energy deficits in the present study on average exceeded both the 500 kcal/day and 600 kcal/day threshold. While it is possible that energy intake was underreported in the present study, it remains plausible that a lack of sufficient energy intake contributed to a lack of significant gain in overall lean/soft tissue mass. Given the high bioenergetic cost of de novo MPS over and above simple regulatory muscle protein turnover [45], participants were likely poorly bioenergetically positioned to accrue lean/soft tissue mass. It is additionally possible that participants experienced hypertrophy only in those areas most heavily recruited by study protocols, namely, the lower extremity. Another consideration in the lack of overall lean/soft tissue mass seen in participants herein is the advanced age of participants (age = 69.7 ± 8.2 years). While it has been shown that adults in the oldest segment of the population (age range = 85–97 years) can achieve gains in fiber cross-sectional area [15], it has additionally been demonstrated via meta-regression that age has an inverse relationship with lean mass gain (β= −0.03; *p* = 0.01) [10]. It is likely that age of participants in this study impacted their achievable lean/soft tissue mass gain from the outset.

The lack of group effects between differential protein intakes is consistent with other research. Ten Haaf et al. report in a recent meta-analysis that protein supplementation while undergoing concomitant RT produced no extra benefit to measures of DXA derived LBM in older adults [67]. Indeed, several studies have reported that protein enhancement strategies (overall diet and/or supplementation) do not lead to lean mass gain when undergoing concomitant RT [14,21,31,68,69,70,71]. While there is evidence to suggest that enhanced protein intake can play a role in the augmentation of lean mass gain in older adults [29,35,72,73], findings to this point remain equivocal, a notion supported by the results of this study. Further research examining the effects of prolonged RT on appendicular and whole-body lean/soft tissue mass, particularly as it relates to functional and clinical outcomes in older adults, is warranted. Additionally, further research examining graded protein intake with sufficient energy intake, and more rigorous nutrient intake measurements are warranted.

Vastus lateralis tissue thickness demonstrated significant between-group differences with the GP group being significantly greater than the CP group. While there is a paucity of literature assessing dietary protein interventions along with RT on vastus lateralis thickness in older adults, it was seen in a study by Aas and colleagues that RT combined with 34 g of a milk protein supplement enhanced ultrasound derived vastus lateralis tissue thickness in a cohort of older adults [74]. Vastus lateralis thickness has been shown both to increase over time with RT in older adults [62,64] and, similar to the present study, show no difference over time with RT [75]. While a significant group * time interaction was not observed for measures of vastus lateralis thickness, it is noteworthy that baseline measures were not significantly different between intervention groups. While this is potentially indicative of a differential improvement due to the graded protein intake intervention, it is also worth noting that neither group demonstrated improvements over time. Further research investigating muscle tissue thickness in response to RT and protein intake in older adults is warranted.

### 4.3. Isokinetic Dynamometry and Muscle Quality Score

Measures of peak torque and muscle quality score at two different velocities responded similarly, with both measures improving over time, but not differentially between groups. Such improvements are in line with several meta-analyses that have shown improvement to muscle quality (defined identically to the present study) [76] and lower body strength [11,16] after a progressive RT intervention. In another study, Brooks et al. reported in a cohort of older (*n* = 62; age ≥ 55 years) adults with type 2 diabetes, those undergoing an RT intervention (16 weeks, 3 sessions/week, 60–80% 1 RM) improved muscle quality and lower body muscle strength to a greater degree than those only receiving standard care (*p* < 0.001) [77]. Additionally, Tracy et al. report that in response to unilateral leg training, muscle quality and 1 RM strength is increased to a greater degree in the trained leg than the untrained leg in a cohort of older adults (age ≥ 65 years) [78]. Notably, these studies defined muscle quality as 1 RM/DXA derived leg lean mass and 1 RM/MRI derived muscle volume respectively.

The reported responses of strength and muscle quality to protein intake modification are variable. At the meta-analytic level, certain findings suggest that enhanced protein intake augments strength gain beyond RT alone [27,73], while another suggests no benefit of enhanced protein intake with RT [35]. At the individual study level, it is commonly reported that strength improves similarly regardless of protein intake status in older adults when an RT intervention is performed [14,17,20,21]. Similarly, certain cross-sectional analyses suggest that enhanced protein intake is beneficial for muscle quality [79,80], while results from a randomized trial in a cohort of elderly women (*n* = 91; mean age = 83.6 years) demonstrate similar improvements to muscle quality between augmented protein intake with RT and RT alone [81]. Given such discordant results, it appears that RT in older adults is the primary driver of improvements in strength and subsequently muscle quality. Indeed, improvements to strength are perhaps the most robust and commonly reported adaptation to RT interventions in older adults. Given the modest, yet significant improvement to appendicular lean/soft tissue mass seen herein and the significance of improvements to muscle quality scores, it appears that improvements to strength outpaced improvement to leg lean/soft tissue mass. This could be due to improvements in neuromuscular adaptation. It has been reported that power output in adults ≥ 65 years declines at a rate of up to 3.5% per annum [82], but RT can improve motor unit discharge rate at maximal force production [83] as well as voluntary agonist activation [84] in older adults. This perhaps provides a basis for the ubiquitous strength increases. Further research examining the precise effects of differential protein intakes in combination with RT on muscle quality is warranted.

### 4.4. Experimental Considerations

Similar to many studies involving intensive RT, the present study is limited to a small sample size. Another limitation of this study is the inability to collect meaningful biological markers of protein accretion or turnover. Given the suggestion that concomitant protein intake and RT is beneficial for inducing anabolic cell signaling events in older adults [26,85], the collection of relevant skeletal muscle signaling markers could have provided a broader picture about what was occurring at the molecular level in these participants. The nature of self-report measures is an additional limitation to this study, as it is apparent that self-report measures do not reflect actual dietary intake with a high degree of certainty [52,53,54,86]. To the authors’ knowledge, however, this was the first study to examine the effectiveness and feasibility of a graded protein intake paradigm in a cohort of older adults. Additionally, this study employed a multifactorial approach to assess aging muscle. A variety of measures aimed at targeting not only muscle mass, but also muscle quality, muscle architecture/morphology, and muscle strength were used.

## 5. Conclusions

In conclusion, the data presented in this preliminary trial suggest that the protein intake and RT paradigm used in this study are feasible in a population of older adults, and further research to examine muscle protein synthesis and degradation using graded protein intake and RT is warranted. Additionally, these data suggest that RT, but not differential protein intake, can improve muscle quality scores at 60°/s and 120°/s and appendicular lean/soft tissue mass in older adults. Vastus lateralis thickness demonstrated the only between-group difference, potentially suggesting that the GP intake intervention is beneficial for improving this measure. Despite ubiquitous improvements across groups, no group * time interactions were observed, suggesting that differential protein intake did not play a role in lean/soft tissue mass, strength, and muscle quality adaptation, provided that 0.8–1.0 g/kg/d of protein is consumed. This study serves as a step to investigate pragmatic interventions to combat the age-related loss of skeletal muscle mass.

## Figures and Tables

**Table 1:** Study design.

	PRE	Wk 0	Wk 1	Wk 2	Wk 3	Wk 4	Wk 5	MID	Wk 6	Wk 7	Wk 8	Wk 9	Wk 10	POST
DXA Scan	X												X	X
Ultrasound	X													X
Dynamometry	X							X						X
Acclimation		X												
Training			X	X	X	X			X	X	X	X		
Deload							X						X	
Nutrition Protocol			X	X	X	X	X		X	X	X	X	X	
3 RM Testing		X												
Training Intensity (% 1 RM)	-	-	60	65	70	75	50	-	70	75	80	85	30	-
Sets/Exercise	-	-	2	2	3	3	2	-	2	3	3	3	1	-
Repetitions/Set	-	-	8–12	8–12	8–12	8–12	8–12	-	8–12	8–12	8–12	8–12	8–12	-
GP Target Protein Intake (g/kg/day)	-	-	0.8	1.0	1.2	1.4	1.4	-	1.6	1.8	2.0	2.2	2.2	-
CP Target Protein Intake (g/kg/day)	-	-	0.8–1.0	0.8–1.0	0.8–1.0	0.8–1.0	0.8–1.0	-	0.8–1.0	0.8–1.0	0.8–1.0	0.8–1.0	0.8–1.0	-
GP Protein Supplement Prescribed (g)	-	-	25	25	25	25	25	-	50	50 or 75 *	50 or 75 *	50 or 75 *	50 or 75 *	-

* Supplemental protein either 50 or 75 g depending on participant preference or failure to meet the previous week’s protein goal. Wk: Week; DXA: Dual Energy X-ray Absorptiometry; RM: Repetition Maximum; CP: Constant Protein Group; GP: Graded Protein Group.

**Table 2:** Participant descriptive characteristics.

Variable	Constant Protein	Graded Protein	Total	*p*-Value
Participant Number	9	9	18	-
Age (Years)	72.11 ± 7.17	67.33 ± 8.93	69.72 ± 8.23	0.229
Sex (Number of males)	3	4	7	0.653
Height (cm)	168.44 ± 8.22	169.06 ± 10.17	168.75 ± 8.98	0.890
Weight (kg)	77.87 ± 19.31	69.47 ± 12.72	73.67 ± 16.44	0.292
Body Mass Index (kg/m^2^)	27.27 ± 5.45	24.31 ± 4.34	25.79 ± 5.02	0.221
Total Lean/Soft Tissue Mass (kg)	45.32 ± 11.24	46.40 ± 8.96	45.86 ± 9.88	0.825
Leg Press Estimated 1 RM (KG)	118.17 ± 32.37	128.05 ± 73.87	123.11 ± 55.56	0.718
Leg Extension Estimated 1 RM (KG)	56.92 ± 15.60	66.04 ± 32.06	61.48 ± 24.90	0.458

**Table 3:** Self-reported energy intake.

	Week	Constant Protein				Graded Protein			
		Absolute (kcals/d)	SD	Relative (kcals/kg/d)	SD	Absolute (kcals/d)	SD	Relative (kcals/kg/d)	SD
Energy (kcal/d or kcal/kg/d)	1	1717	359	22.58	4.11	1594	458	23.16	5.35
	2	1698	220	22.66	4.54	1601	436	23.05	4.05
	3	1508	242	20.17	4.89	1665	439	23.95	4.17
	4	1474	292	20.12	6.38	1864 #	431	27.17 #	5.42
	5	1555	309	20.44	3.63	1887 #	275	27.47 #	2.68
	6	1655	238	22.05	4.52	1822	428	26.43	4.74
	7	1580	187	21.15	4.32	1939	590	28.21 #	7.46
	8	1573	155	21.17	4.73	2013	667	29.21#	8.18
	9	1484	342	19.57	4.26	2259 *#	578	32.86 *#	7.04
	10	1623	297	21.22	2.20	2240 *#	700	32.32 *#	7.53

* Significantly different from Week 1, # Significantly different from CP group. kcals: kilocalories; d: days.

**Table 4:** Self-reported protein intake.

	Week	Constant Protein				Graded Protein			
		Absolute (g/d)	SD	Relative (g/kg/d)	SD	Absolute (g/d)	SD	Relative (g/kg/d)	SD
Protein (g/d or g/kg/d)	1	73	16	0.96	0.11	80	22	1.18	0.33
	2	77	16	1.00	0.15	81	23	1.16	0.24
	3	72	15	0.94	0.13	86	15	1.25 #	0.16
	4	69	15	0.91	0.15	98 #	17	1.41 #	0.08
	5	72	16	0.94	0.07	99 #	18	1.42 #	0.05
	6	79	21	1.01	0.08	116 *#	18	1.69 *#	0.15
	7	72	15	0.93	0.09	125 *#	21	1.81 *#	0.13
	8	71	16	0.94	0.21	134 *#	26	1.93 *#	0.13
	9	74	17	0.96	0.07	152 *#	27	2.20 *#	0.08
	10	75	19	0.97	0.18	150 *#	26	2.17 *#	0.16

* Significantly different from Week 1, # Significantly different from CP group; d: days.

**Table 5:** Graded protein group prescribed protein intake vs. actual protein intake.

Week	1	2	3	4	5	6	7	8	9	10
Target Protein Intake (g/day)	0.8	1.0	1.2	1.4	1.4	1.6	1.8	2.0	2.2	2.2
Actual Protein Intake (g/day)	1.17	1.16	1.25	1.41	1.42	1.69	1.81	1.93	2.20	2.17
% Difference	37.92	15.10	4.43	0.40	1.42	5.41	0.55	−3.45	−0.10	−1.58

**Table 6:** Summary of effects, calculated power, and estimated sample size for each measured variable.

Outcome	Main Effect/Interaction	*p*-Value	F-Value	η_p_^2^	Observed Power (%) *	Estimated Sample Size *
Total Lean/Soft Tissue Mass	Time	0.245	1.457	0.083	66.9	24
	Group	0.867	0.029	0.002	5.5	2940
	G * T	0.209	1.714	0.097	74.3	22
Appendicular Lean/Soft Tissue Mass	Time	0.028	5.819	0.267	99.7	8
	Group	0.974	0.001	<0.001	-	-
	G * T	0.634	0.236	0.015	16.6	132
Lean/Soft Tissue Mass Index	Time	0.281	1.244	0.072	60.3	28
	Group	0.838	0.043	0.003	5.7	1960
	G * T	0.207	1.729	0.098	74.7	22
Vastus Lateralis Thickness	Time	0.455	0.586	0.035	33.0	58
	Group	0.040	5.003	0.238	72.9	22
	G * T	0.384	0.802	0.048	43.3	42
Knee Extensor Peak Torque 60°/s	Time	<0.001	10.218	0.390	99.9	6
	Group	0.199	1.797	0.101	33.9	56
	G * T	0.225	1.564	0.089	70.2	24
Knee Flexor Peak Torque 60°/s	Time	<0.001	23.623	0.596	99.9	6
	Group	0.588	0.305	0.019	9.8	306
	G * T	0.293	1.277	0.074	61.5	28
Knee Extensor Peak Torque 120°/s	Time	0.004	6.608	0.292	99.9	8
	Group	0.197	1.814	0.102	34.2	54
	G * T	0.514	0.679	0.041	37.8	48
Knee Flexor Peak Torque 120°/s	Time	<0.001	12.155	0.432	99.9	6
	Group	0.570	0.336	0.021	10.4	278
	G * T	0.782	0.247	0.015	16.6	132
Muscle Quality Score 60°/s	Time	0.001	16.052	0.501	100.00	6
	Group	0.198	1.802	0.101	33.9	56
	G * T	0.152	2.263	0.124	85.0	18
Muscle Quality Score 120°/s	Time	0.015	7.408	0.316	99.9	8
	Group	0.271	1.298	0.075	25.9	76
	G * T	0.444	0.616	0.037	34.6	54

* Observed power and estimated sample size only calculated for η_p_^2^ values > 0.001.

## Data Availability

Data from this study are available from the principal investigator upon reasonable request.
